# Blocking hyaluronan synthesis alleviates acute lung allograft rejection

**DOI:** 10.1172/jci.insight.142217

**Published:** 2021-11-22

**Authors:** Jewel Imani, Kaifeng Liu, Ye Cui, Jean-Pierre Assaker, Junwen Han, Auyon J. Ghosh, Julie Ng, Shikshya Shrestha, Anthony M. Lamattina, Pierce H. Louis, Anne Hentschel, Anthony J. Esposito, Ivan O. Rosas, Xiaoli Liu, Mark A. Perrella, Jamil Azzi, Gary Visner, Souheil El-Chemaly

**Affiliations:** 1 Division of Pulmonary and Critical Care Medicine, Brigham and Women’s Hospital, Harvard Medical School, Boston, Massachusetts, USA.; 2 Division of Pulmonary and Critical Care Medicine, Boston Children Hospital, Harvard Medical School, Boston, Massachusetts, USA.; 3 Transplantation Research Center, Renal Division, and; 4 Department of Pediatric Newborn Medicine, Brigham and Women’s Hospital, Harvard Medical School, Boston, Massachusetts, USA.; 5 Division of Pulmonary, Critical Care, and Sleep Medicine, SUNY Upstate Medical University, Syracuse, New York, USA.; 6 Pulmonary, Critical Care and Sleep Medicine, Baylor College of Medicine, Houston, Texas, USA.

**Keywords:** Pulmonology, Organ transplantation

## Abstract

Lung allograft rejection results in the accumulation of low–molecular weight hyaluronic acid (LMW-HA), which further propagates inflammation and tissue injury. We have previously shown that therapeutic lymphangiogenesis in a murine model of lung allograft rejection reduced tissue LMW-HA and was associated with improved transplant outcomes. Herein, we investigated the use of 4-Methylumbelliferone (4MU), a known inhibitor of HA synthesis, to alleviate acute allograft rejection in a murine model of lung transplantation. We found that treating mice with 4MU from days 20 to 30 after transplant was sufficient to significantly improve outcomes, characterized by a reduction in T cell–mediated lung inflammation and LMW-HA content and in improved pathology scores. In vitro, 4MU directly attenuated activation, proliferation, and differentiation of naive CD4^+^ T cells into Th1 cells. As 4MU has already been demonstrated to be safe for human use, we believe examining 4MU for the treatment of acute lung allograft rejection may be of clinical significance.

## Graphical abstract

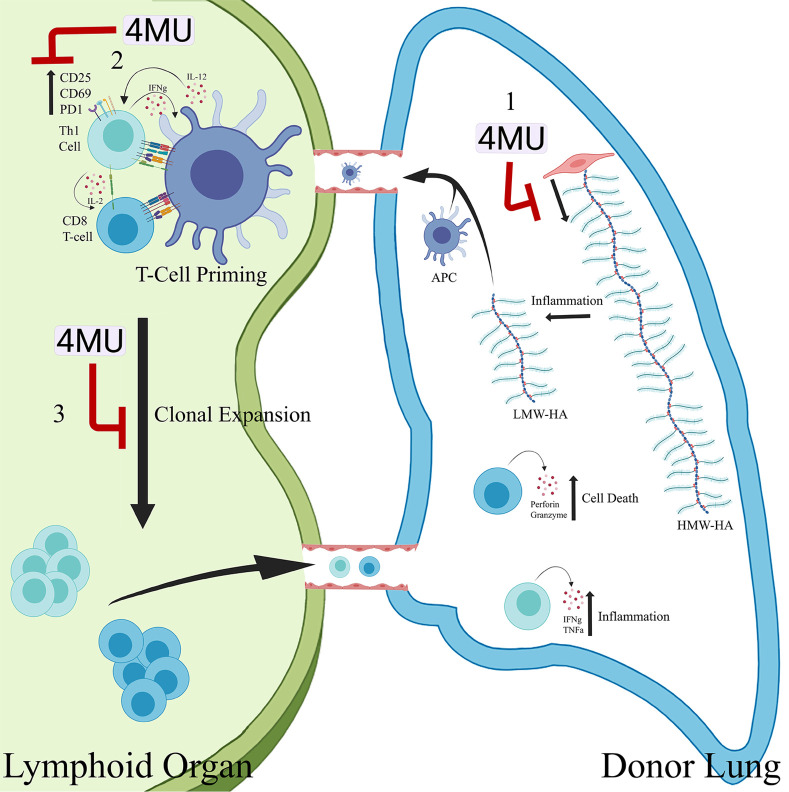

## Introduction

Lung transplantation is the only therapy for end-stage lung disease, resulting in an improvement in overall quality of life and life expectancy (1). Every year, there are 4500–5000 patients who receive lung transplants worldwide, approximately 28% of whom experience acute rejection in their first postoperative year and have a 5-year survival of only 50% (2). Acute lung allograft rejection is an important risk factor for chronic lung allograft rejection and graft failure. Acute rejection causes damage to endothelial and epithelial cells of the donor tissues and activates nonspecific innate responses, which lead to allograft-specific adaptive responses by host T cells and B cells (3, 4). Upon activation, T cells clonally expand, differentiate into effector cells, and subsequently migrate to sites of inflammation (e.g., the donor lung graft). CD4^+^ T cells can differentiate into either Th 1 or Th2; Th1 cells are primarily associated with acute cellular rejection and produce IFNG, TNFA, and IL-2 that can stimulate the humoral response, increase expression of MHC on DCs, and prime CD8^+^ T cells, while CD8^+^ T cells primed by both DCs and Th1 cells (5) differentiate into cytotoxic T cells that directly kill target donor cells via secretion of perforin and granzyme B or cell-to-cell contact via the FAS/FAS ligand (FAS/FASL) pathway and are major contributors to graft rejection in murine models of allograft transplantation (6). At the donor tissue site, effector T cells can also activate other immune cells, including macrophages, neutrophils, eosinophils, and basophils. In addition to T cell–mediated inflammation, there are various other proinflammatory mediators in the lungs, including low–molecular weight hyaluronic acid (LMW-HA), a potent inducer of inflammation. HA is an extracellular matrix (ECM) glycosaminoglycan that is ubiquitously expressed throughout the lung tissue and is synthesized under the control of 3 HA synthase (HAS) isozymes (HAS1, HAS2, and HAS3) (7). Under physiological conditions, high–molecular weight HA (HMW-HA) can polymerize up to 2 × 10^4^ kDa in size (8, 9). As a result of its large size and physical-chemical properties, HMW-HA is markedly hygroscopic, modulating both the osmotic balance and hydration of the lung tissues. Furthermore, it provides structural support to the ECM by acting as scaffolding for proteoglycans, fibrinogen, collagen, and elastin fibers to adhere to. It also binds to cells and modulates cell migration, proliferation, and differentiation (10). During homeostasis, HA is continually turned over and fragmented by hyaluronidases and mechanical forces (11) into various-sized polymers, including LMW-HA, which is normally primarily cleared by the lymphatics (12) and alveolar macrophages (13). However, during injury and inflammation, HA levels significantly rise, are further fragmented by reactive oxygen species, and predominately exist as LMW-HA polymers (14, 15). LMW-HA can bind and activate DCs, monocytes, and macrophages through TLR2 and TLR4 signaling (16–19), modulating their migratory and trafficking patterns (20, 21), as well as promoting the release of chemokines and proinflammatory cytokines (22, 23). In the context of lung transplantation, we and others have shown that LMW-HA is a potent inducer of tissue inflammation and is associated with the development of acute and chronic rejection (17, 24, 25). During homeostasis, up to 85% of HA is cleared through lymphatic drainage, and we have previously shown that induction of lymphangiogenesis between days 20 and 30 after transplant reduced LMW-HA in the lungs and was associated with a reduction of lung rejection (25). We, therefore, asked if modulation of HA levels in the lungs could improve post–lung transplant outcomes. 4-Methylumbelliferone (4MU), a known inhibitor of HA synthesis (26–30), has been shown to reduce expression of HAS1, HAS2, and HAS3 gene expression (28, 31, 32); it also acts as a competitive substrate for the enzyme UDP-glucuronosyltransferase (UGT) and ligates to glucuronic acid, thereby limiting its availability to incorporate into growing HA polymers.

We, therefore, hypothesized that treatment with 4MU following lung transplantation in a murine model could improve outcomes from allograft rejection. Briefly, we performed orthotopic single (left) lung transplants across MHC-mismatched strains of mice (BALB/c into C57BL/6) and treated the recipients with 4MU or vehicle from days 20 to 30 after transplant. We found that treatment with 4MU improved posttransplant outcomes, and this improvement was associated with reduced levels of LMW-HA in the lungs. 4MU treatment also reduced the number of memory T cells in vivo and prevented the proliferation and polarization of in vitro–activated CD4^+^ T cells into IFNG-producing Th1 cells.

## Results

### 4MU treatment ameliorates orthotopic lung transplant rejection.

**Figure 1:**
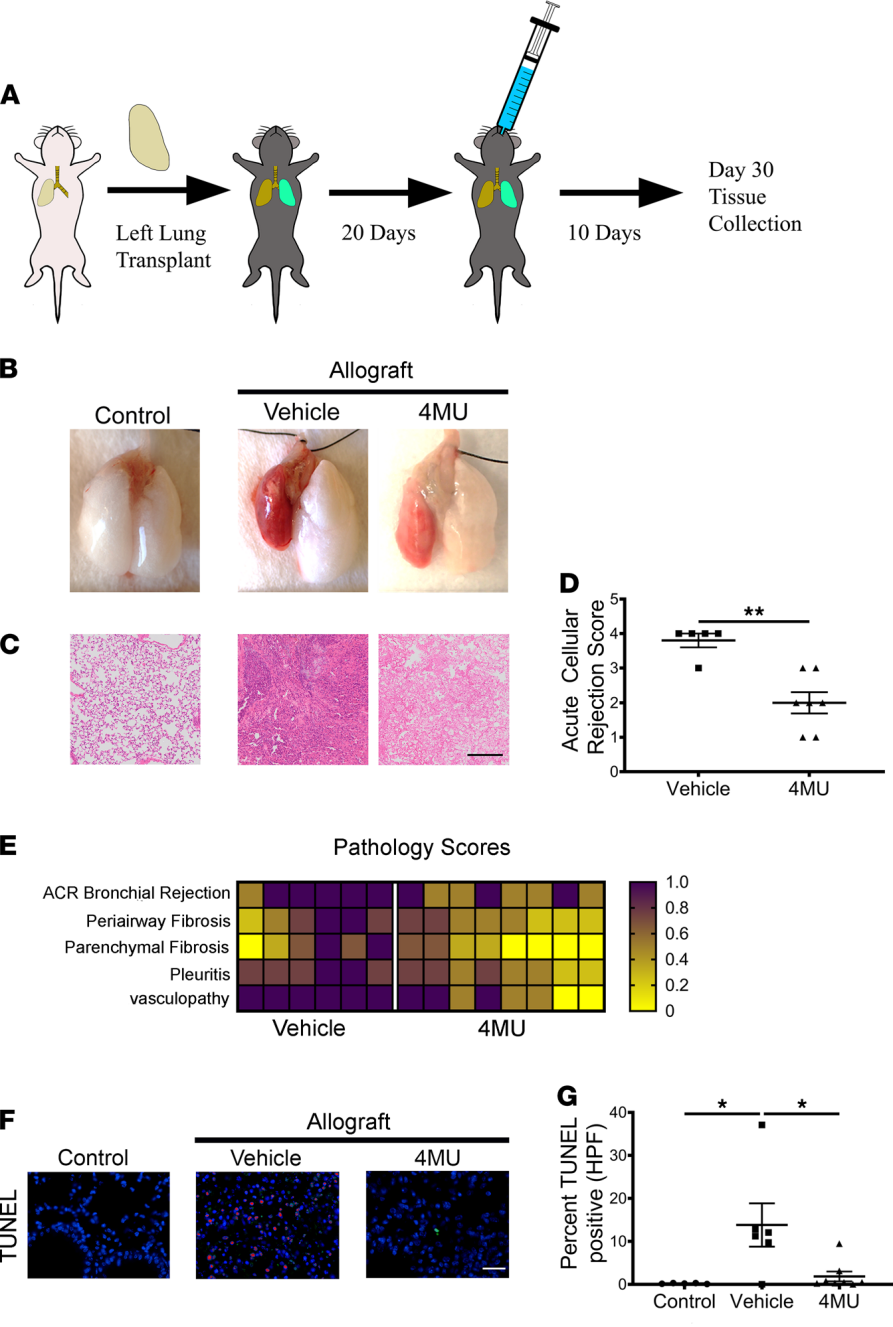
Treatment with 4MU ameliorates lung transplant rejection. Left lungs from donor BALB/c mice were orthotopically transplanted into recipient C57BL/6 mice on day 0 (**A**). Starting at day 20 after transplant, the recipient mice were gavaged daily with 4MU (450 mg/kg, 1% Arabic gum in PBS). On day 30 after transplant, the recipient mice were sacrificed, and tissues were collected for analysis. Schematic diagram of the transplant and treatment procedure (**A**). Representative gross pathology of control (*n* = 6), untreated allograft transplanted (*n* = 6), or 4MU-treated allografts (*n* = 8). Transplanted grafts are depicted on the left side of each lung block (**B**). Representative images from donor left lung sections stained with H&E and imaged at 200× magnification. Scale bar: 200 μm (**C**). Acute cellular rejection scores of vehicle (*n* = 5) and 4MU-treated mice (*n* = 7) (**D**). Heatmap of lung pathology scores (**E**). Representative images of TUNEL^+^ apoptotic cells (pink) from donor left lungs. Scale bar: 20 μm (**F**). Quantitative analysis of TUNEL^+^ apoptotic cells from 5 random high-power fields from control (*n* = 5), vehicle-treated (*n* = 5), and 4MU-treated (*n* = 5) mice (**G**). Data are represented as mean ± SEM and analyzed using 1-way ANOVA followed by Tukey’s post hoc test for multiple comparisons (*P < 0.05) or 2-tailed Student’s *t* test (**P < 0.01).

To examine the effects of 4MU on outcomes following lung transplantation, orthotopic single-lung transplants were carried out across MHC-mismatched strains of mice (BALB/c H2^D^ -> C57BL/6 H2^B^) (33). Recipient mice received 4MU (450 mg/kg) or vehicle by oral gavage starting at day 20 after transplant until study endpoint at day 30 (Figure 1A). Compared with control mice transplanted with an isograft donor lung, mice transplanted with allografts and treated with vehicle had noticeably worse posttransplant outcomes (Figure 1B) grossly characterized by reduced lung size (compared with native right lung) and generalized diffuse erythema. In contrast, mice transplanted with lung allografts and treated with 4MU had improved lung morphology. Histological examination (Figure 1C) of H&E-stained lung tissues revealed significant inflammation in vehicle-treated mice, whereas mice treated with 4MU had reduced inflammation and edema. Examination of the recipient’s right lungs (Supplemental Figure 1; supplemental material available online with this article; https://doi.org/10.1172/jci.insight.142217DS1) revealed that they were histologically intact. Blind classification and quantification of the acute cellular rejection score by a pulmonologist (Figure 1D) indicated that 4MU-treated mice had significantly reduced cellular rejection (*P* < 0.01) compared with mice treated with vehicle. Further classification of H&E and Masson’s trichome stained lungs (Figure 1E and Supplemental Figure 2) revealed that, compared with 4MU-treated mice, untreated mice had worse posttransplant outcomes characterized by increased parenchymal fibrosis, thick pleuritis, and fibrosis compared with 4MU-treated mice.

Next, we assessed whether 4MU treatment affected cell death of lung cells by using a TUNEL assay to stain for apoptotic cells in lung sections derived from the donor. As expected, we observed positive TUNEL staining in mice treated with vehicle. In contrast, there was a significant decrease in TUNEL^+^ cells in mice treated with 4MU (Figure 1, F and G). Collectively, these data demonstrate that treatment with 4MU in post–lung transplant recipient mice reduces acute transplant rejection. 

### 4MU treatment reduces HA levels in the lung.

**Figure 2:**
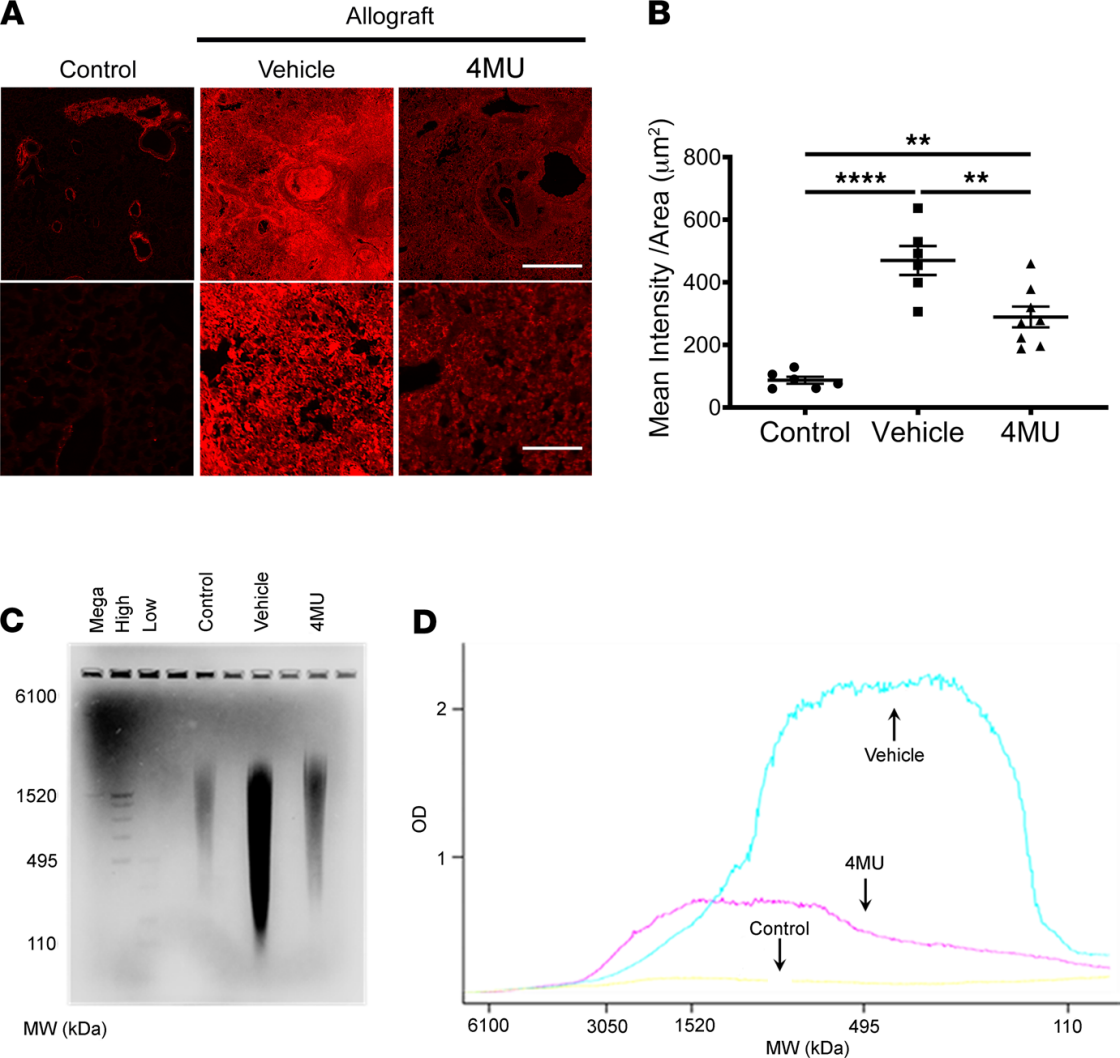
Treatment with 4MU modulates the synthesis of hyaluronic acid. Representative images of donor left lung sections stained with biotinylated HABP followed with streptavidin-labeled Texas Red fluorescent dye. Scale bar: 500 μm top image, 100 μm bottom image (**A**). Mean intensity of fluorescently stained lung sections from control (*n* = 6), vehicle-treated (*n* = 6), and 4MU-treated (*n* = 8) mice (**B**). Digested lungs from the experimental groups were pooled and loaded onto a 0.7% agarose gel and stained for HA (**C**). Quantitative analysis of HA molecular mass profile by measuring the Optical density (OD) of the agarose gel (**D**). Data are represented as mean ± SEM and analyzed using 1-way ANOVA followed by Tukey’s post hoc test for multiple comparisons. **P < 0.01; ****P < 0.0001.

To examine the levels of HA in the lungs, 5 μm lung tissue sections were stained with biotinylated HA binding protein (HABP), followed by secondary staining with Texas Red–conjugated streptavidin; they were subsequently imaged (Figure 2A). Compared with control isograft transplanted mice, transplantation of allograft lungs significantly increased HA staining in the lungs (*P* < 0.0001) (Figure 2B). However, when recipient mice were treated with 4MU, HA levels were significantly reduced compared with the vehicle group (*P* < 0.01) but not back to the level of the control mice transplanted with isografts (*P* < 0.01). We next examined whether 4MU treatment modulates the molecular size distribution of HA; agarose gel electrophoresis of pooled lung lysates (Figure 2C) was used to determine the distribution of HMW-HA and LMW-HA. Control mice transplanted with isograft lungs had an even distribution of HA molecular size throughout the measured range (6100–110 kDA) with an OD of less than 1 (Figure 2D). In contrast, the distribution of HA molecular size in vehicle-treated mice shifted to the right within the range of 1520–110 kDa with an associated increase in OD above 2, indicating more LWM-HA. This increase in LMW-HA was ameliorated when mice transplanted with allograft lungs were treated with 4MU; these mice had an HA OD value of less than 1 throughout the measured range, indicating that 4MU treatment substantially reduced the level of proinflammatory LMW-HA following allogeneic lung transplantation. Taken together, treatment with 4MU in mice transplanted with allograft lungs reduces proinflammatory LMW-HA compared with mice treated with vehicle only.

### 4MU treatment modulates the immune response in allograft rejection.

**Figure 3:**
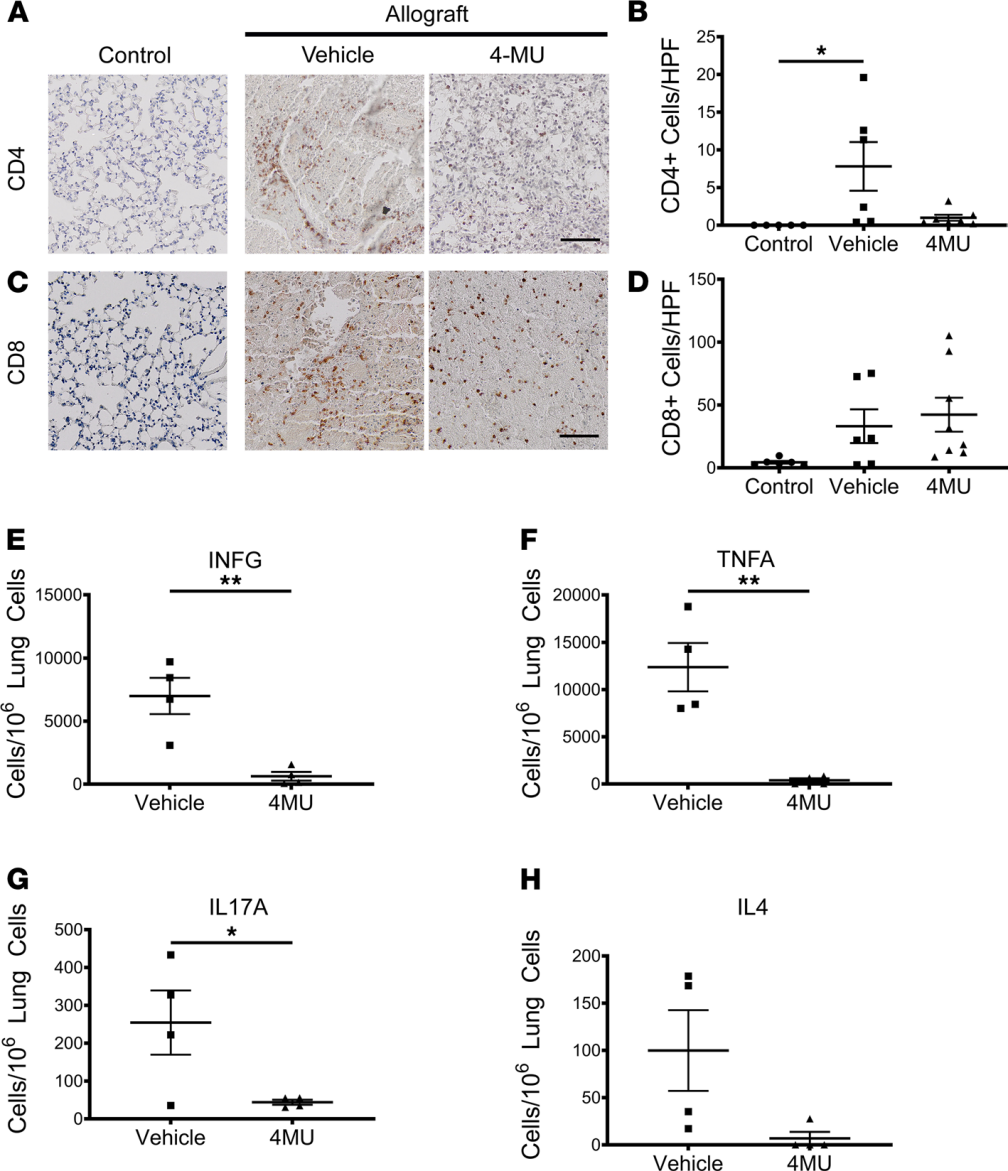
Inhibition of hyaluronic acid synthesis reduces posttransplant cellular rejection. Representative images of donor left lungs stained with anti–mouse CD4 (**A**) and CD8 (**C**) followed by DAB chromogen IHC secondary stain and hematoxylin counterstain. Scale bar: 100 μm. Quantitative analysis of CD4 (**B**) and CD8 (**D**) DAB^+^ cells from 5 random high-powered fields per mouse lung from control (*n* = 6), vehicle (*n* = 6), 4MU (*n* = 8) treated mice. Single-cell suspensions of lung tissues from vehicle-treated (*n* = 4) and 4MU-treated (*n* = 4) (450 mg/kg 5% Arabic gum in PBS) mice were stained against CD3E, CD4, and IFNG (**E**), TNFA (**F**), IL-17A (**G**), and IL-4 (**H**) (flow gating strategy shown in Supplemental Figure 3). Data are represented as mean ± SEM and analyzed using 1-way ANOVA followed by Tukey’s post hoc test for multiple comparisons (*P < 0.05) or 2-tailed Student’s *t* test (*P < 0.05, **P < 0.01).

Following the observation that 4MU treatment reduces posttransplant acute rejection, we asked if there was a modulation of the immune response. To assess the immune status of the lung, we stained 5 μm lung sections with anti-CD4 and anti-CD8 to assess changes in the T cell profile. We only observed CD4^+^ cells in mice transplanted with allograft lungs, but there was a significant increase in the vehicle group compared with 4MU-treated mice (*P* < 0.05) (Figure 3, A and B). Like CD4, we did not observe significant CD8^+^ staining in mice transplanted with isografts, but we did see substantially more CD8^+^ staining in mice transplanted with allograft lungs. However, there was no significant difference between vehicle-treated and 4MU-treated groups (Figure 3, C and D). To further characterize changes in T cell populations, we stained single-cell suspensions of lung cells against CD3E, CD4, CD8A, IFNG, TNFA, IL-17A, and IL-4 and analyzed the cells using flow cytometry (gating strategy in Supplemental Figure 3). We first noted that the IHC analysis may have underestimated the numbers of CD4^+^ cells since the flow data show comparable numbers of CD4^+^ and CD8^+^ cells. However, similar to the immunostaining, there was a decrease in CD4^+^ cells (53,000 ± 10,700 versus 9600 ± 2800, *P* < 0.01) but not a decrease in CD8^+^ cells after 4MU treatment (25,000 ± 8300 versus 10,500 ± 3400, NS). In the vehicle group, we observed a significant number of IFNG^+^ and TNFA^+^ cells within the CD3^+^CD4^+^ population (Figure 3, E and F). However, we did not observe significant numbers of IL-17A^+^ and IL-4^+^ cells (compared with IFNG^+^ and TNFA^+^ cells) (Figure 3, G and H), suggesting that the CD4 helper response at day 30 after transplant was predominantly mediated by Th1 cells. Examination of these cell populations in the 4MU-treated mice revealed a significant decrease in the number of IFNG^+^ (*P* < 0.01), TNFA^+^ (*P* < 0.01), and IL-17A^+^ (*P* < 0.05) cells. Importantly, administration of 4MU did not result in changes in IL-4^+^CD4^+^ T cells. Within the CD8^+^ T cell population, 4MU treatment did not significantly change the number total CD8 T cells or IFNG^+^ and TNFA^+^ cells (not shown).

To further elucidate changes in proinflammatory cytokines, we extracted bulk mRNA from transplanted donor lungs and quantified cytokines via quantitative PCR (qPCR). We observed a 7-fold increase in *Ifng*, a 2-fold increase in *tnfa*, and a 3-fold significant increase in *Ccr5* between isograft and allograft transplanted mice treated with vehicle (*P* = 0.0001) (Supplemental Figure 4). This phenotype is consistent with a Th1 immune response. In the mice treated with 4MU, *Ifng* mRNA was no longer detectable in the lungs, whereas *Tnf* and *Ccr5* (*P* < 0.0001) expression was reduced to levels observed in the control isograft–transplanted mice. Collectively, these data demonstrate there was a type 1 CD4^+^ T cell response at day 30 in the mice transplanted with allogeneic donor tissues, and treatment with 4MU attenuated that response.

### 4MU inhibits the development of memory T cells.

**Figure 4:**
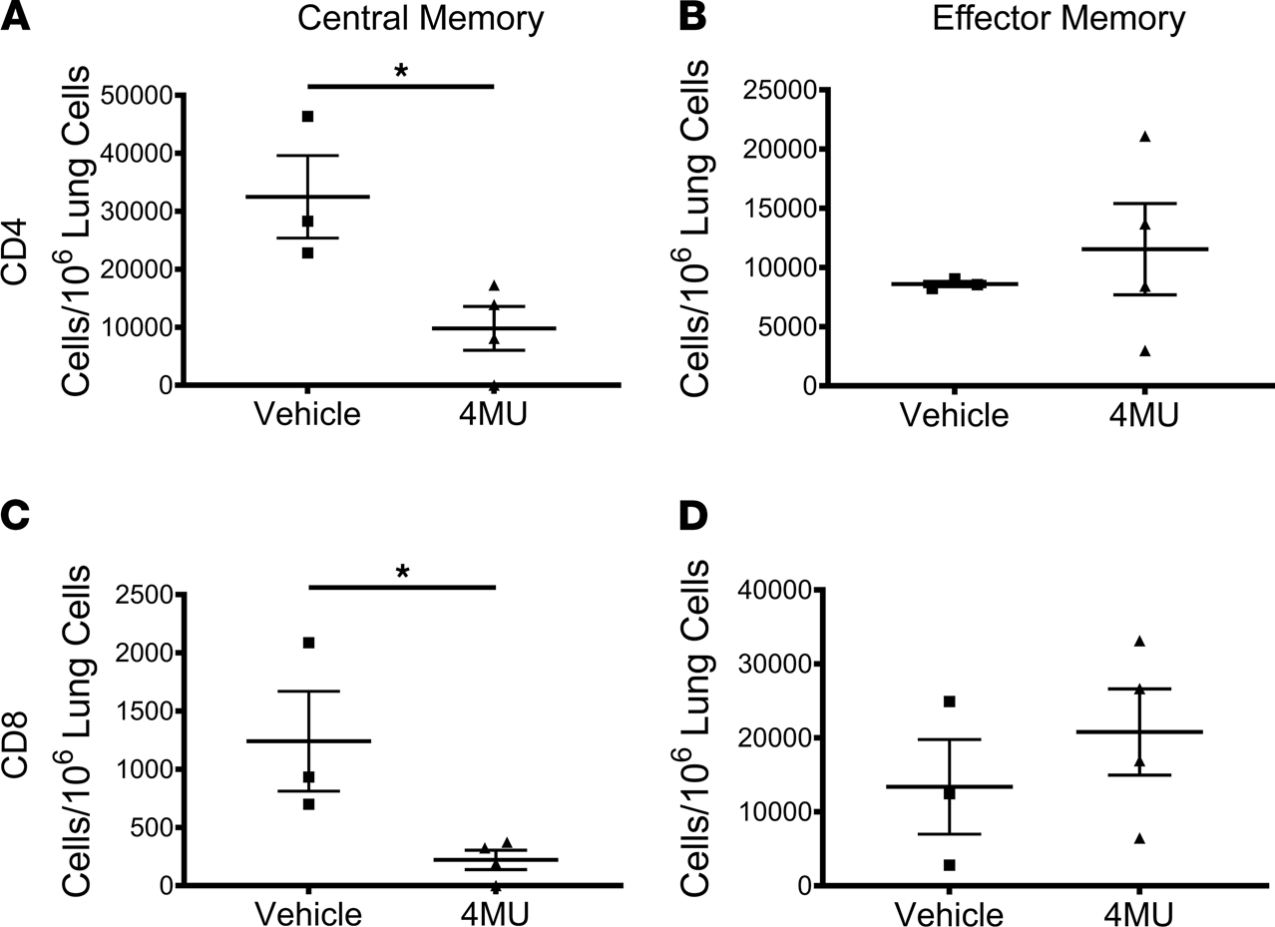
Inhibition of hyaluronic acid synthesis reduces the formation of memory T cells. Single-cell suspension prepared from lungs of mice treated with vehicle-treated (*n* = 3) or 4MU-treated (*n* = 4) (450 mg/kg 5% Arabic gum in PBS) mice were stained for CD4^+^CD62L^+^CD44^+^ central memory T cells (**A**), CD4^+^62L-CD44^+^ effector memory T cells (**B**), CD8^+^CD62L^+^CD44^+^ central memory T cells (**C**), and CD8^+^62L-CD44^+^ effector memory T cells (**D**) (flow gating strategy shown in Supplemental Figure 5). Data are represented as mean ± SEM and analyzed using 2-tailed Student’s *t* test. *P < 0.05.

Since memory T cell responses are a major hurdle to overcome in inducing transplant tolerance (34, 35), we next asked whether 4MU can modulate memory T cell responses following allogeneic lung transplantation. To investigate, the lungs from transplanted mice were harvested on day 30 after transplant and analyzed by flow cytometry to determine the number of CD4^+^ and CD8^+^ central memory (CD44^+^CD62L^+^) (Figure 4, A and C, and Supplemental Figure 5) and effector memory (CD44^+^CD62L^–^) (Figure 4, B and D) T cells. In mice transplanted with allograft lungs and treated with 4MU, there was a significant decrease in the number of CD4^+^ central memory T cells (*P* < 0.05) compared with vehicle-treated mice. 4MU treatment similarly reduced the number of CD8^+^ central memory T cells (*P* < 0.05). In contrast, there was no difference in the number of CD4^+^ or CD8^+^ effector memory T cells between the vehicle or 4MU-treated mice. These data suggest that 4MU reduces the transition of T cells into memory T cells.

### 4MU inhibits effector T cells in vitro.

**Figure 5:**
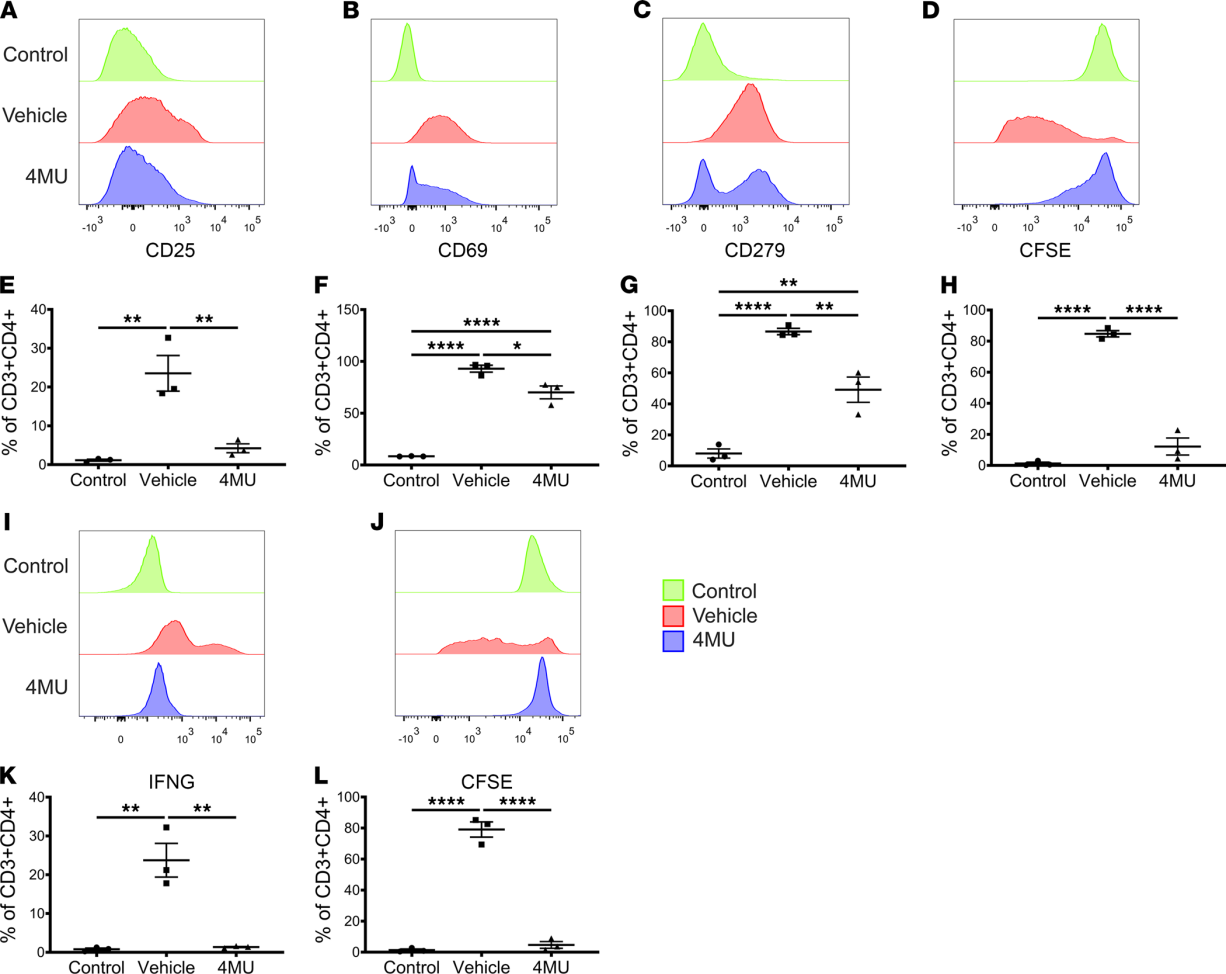
4MU blocks activation and polarization of naive CD4 T cells into Th1 cells. CFSE-stained naive CD4^+^ T cells from C57BL/6 mice (*n* = 3) were stimulated for 5 days with anti-CD3E, anti-CD28, or rhIL-2 ± 4MU (100 μg/mL) and then flow analyzed to quantify expression of CD25 (**A** and **E**), CD69 (**B** and **F**), CD279 (**C** and **G**), and CFSE dilution (**D** and **H**). CFSE-stained naive CD4^+^ T cells from C57BL/6 mice (*n* = 3) were stimulated and polarized toward Th1 cells for 5 days with anti-CD3E, anti-CD28, rhIL-2, rmIL-12, or anti–IL-4 ± 100 μg/mL 4MU and then flow analyzed for expression and quantification of IFNG (**I** and **K**) and CFSE dilution (**J** and **L**) (flow gating strategy shown in Supplemental Figures 6 and 7). Data are represented as mean ± SEM and analyzed using 1-way ANOVA followed by Tukey’s post hoc test for multiple comparisons. *P < 0.05; **P < 0.01; ***P < 0.001; ****P < 0.0001.

To address the possibility that 4MU can inhibit the activation of naive T cells into effector T cells, we isolated and stimulated CFSE-labeled naive C57BL/6 CD4^+^ T cells in vitro with 10 μg/mL anti–mouse CD3E, 2 μg/mL anti–mouse CD28, and 40 U/mL rhIL-2 in the presence of 100 μg/mL 4MU or DMSO (vehicle). After 5 days of stimulation, the T cells were collected and analyzed by flow cytometry (Supplemental Figure 6) for expression of CD25, CD69, CD279 (PD1), and CFSE dilution. Compared with naive control CD4^+^ T cells, stimulated T cells treated with vehicle had a significant increase in CD25 (*P* < 0.01) (Figure 5, A and E) and CD69 expression (*P* < 0.0001) (Figure 5, B and F). Furthermore, the stimulated T cells expressed significant levels of CD279 (*P* = 0.0001) (Figure 5, C and G) compared with unstimulated naive cells, indicating that the T cells were sufficiently stimulated and activated. Collectively, the expression profile suggests that these T cells were both activated and actively proliferating. Indeed, an examination of CFSE staining in these cells revealed that they had undergone multiple rounds of cell division, with ~85% (*P* < 0.0001) of the T cells having undergone at least 1 round of cell division (Figure 5, D and H). In contrast, when T cells were stimulated in the presence of 4MU (100 μg/mL), there was a significant reduction in CD25 (*P* < 0.01) and CD69 expression (*P* < 0.05) compared with vehicle-treated cells. There was no difference in CD25 expression between the unstimulated naive cells and the 4MU treatment groups, but CD69 expression in the 4MU group remained elevated compared with the unstimulated cells (*P* > 0.05) (Figure 5, E and F). 4MU treatment also reduced the expression of CD279 (*P* < 0.01) and blocked cellular division (*P* < 0.0001) (Figure 5, G and H). Collectively, our in vitro data suggest that 4MU reduces but does not completely block T cell activation in the context of CD69 expression, but it blocks the expression of CD25 and subsequently inhibited T cell proliferation.

### 4MU inhibits Th1 differentiation in vitro.

Since we observed a reduction in Th1 CD4^+^ T cells in the lungs following 4MU treatment, we investigated if 4MU could specifically inhibit Th1 polarization; we stimulated CFSE stained naive C57BL/6 CD4^+^ T cells in vitro with 10 μg/mL anti–mouse CD3E, 2 μg/mL anti–mouse CD28, 40 U/mL rhIL-2, 10 ng/mL rmIL-12, and 10 μg/mL anti–mouse IL-4 in the presence of 100 μg/mL 4MU or DMSO (vehicle). After 5 days of incubation, the cells were collected and analyzed for IFNG expression and CFSE dilution using flow cytometry (Supplemental Figure 7). Compared with unstimulated control cells, stimulated T cells significantly increased expression of the Th1 cytokine IFNG (*P* < 0.01) (Figure 5, I and K), which was associated with increased cellular proliferation (*P* < 0.0001) (Figure 5, J and L). In contrast, when T cells were stimulated in the presence of 100 μg/mL 4MU, they neither expressed IFNG nor underwent proliferation. In summary, 4MU inhibits activation of naive CD4^+^ T cells and blocks polarization into IFNG-producing Th1 type cells.

### Differential expression and pathway analysis.

**Figure 6:**
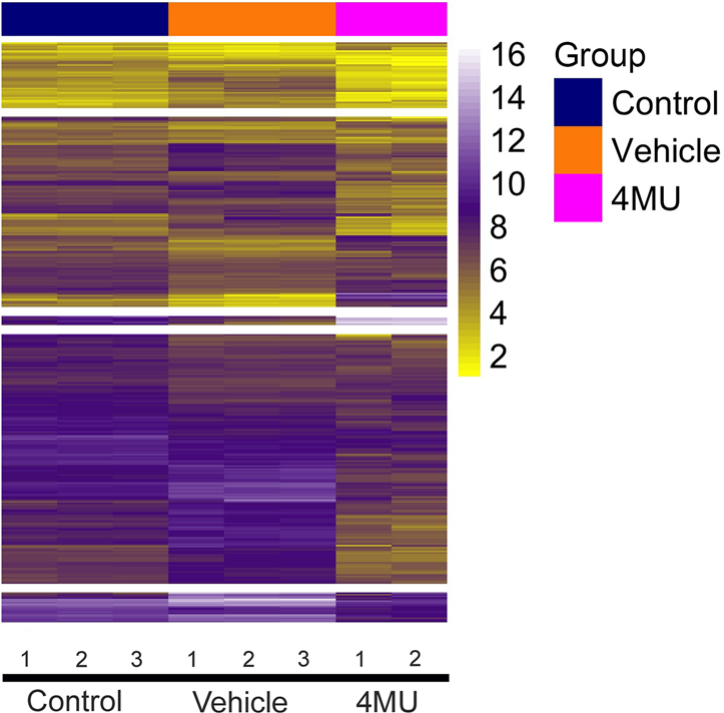
Heatmap cluster and KEGG canonical pathways. Naive CD4^+^ T cells from C57BL/6 mice (*n* = 3) were stimulated for 5 days in vitro with anti-CD3E, anti-CD28, or rhIL-2 ± 4MU (100 μg/mL). RNA was collected and sequenced from control (*n* = 3), stimulated (*n* = 3), and 4MU-treated (100 μg/mL) (*n* = 2) wells. Heatmap of top 369 differentially regulated genes between the groups.

To gain more insights into mechanisms of T cell activation and the effects of 4MU, we stimulated naive C57BL/6 CD4^+^ T cells with 4MU or DMSO (as previously described) and collected RNA for sequencing after 5 days from 9 samples (3 naive, 3 stimulated, and 3 treated with 4MU). One 4MU-treated sample was excluded from differential expression analysis due to poor quality/outlier results. Following preanalysis filtering, we analyzed 1264 genes for differential expression across the 3 treatment groups. We found 369 genes that were differentially expressed with an FDR < 10%. The log-transformed counts for the 369 differentially expressed genes in the 8 samples were clustered using *k* means clustering (Figure 6). After specifying 3 clusters, the samples were clustered by treatment group. The genes were clustered into 5 groups. In clusters 1, 2, 4, and 5, the 4MU treatment group had the lowest expression, followed by the naive group and then the stimulated group with the highest expression. In contrast, in cluster 3, the stimulated group had the lowest expression, followed by the control group and then the treatment group with the highest expression. Pathway analysis was then performed with the KEGG canonical pathways and Gene Ontology Biological Processes (GO-BP) pathway sets. We selected the top 10 pathways by nominal *P* value in each pathway set enriched for the 369 differentially expressed genes. The top 10 KEGG canonical pathways (Table 1) include the TLR signaling pathway (*P* < 0.01) and the chemokine signaling pathway (*P* < 0.01). The top 10 GO biological processes include the response to TNF pathway (*P* < 0.0001) and the lymphocyte chemotaxis pathway (*P* < 0.001) (Supplemental Table 1).

## Discussion

In this study, we demonstrate that the accumulation of LMW-HA fragments is associated with tissue injury and acute allograft rejection. We also show that inhibition of HA synthesis results in the decrease of Th1 CD4^+^ T cells at day 30 after transplant, and this was associated with an improved outcome following orthotopic lung transplantation. These findings highlight the importance of targeting HA as a potential therapeutic modality for acute allograft rejection.

Mouse models of orthotopic lung transplantation have shown that CD8^+^ T cells are sufficient to induce allograft rejection as early as 7 days after transplant; CD4^+^ T cells have also been implicated but have been reported to be not necessary for acute rejection (36–40). Herein, we show that there is an infiltration of type 1 CD4^+^ T cells in the lungs at day 30 after transplant. Following 4MU treatment from days 20 to 30 after transplant, we observed a significant decrease in the levels of total HA and LMW-HA in the lungs. The binding of HA to CD44 on activated T cells has been shown to promote migration and extravasation of effector T cells from the vascular supply into sites of inflammation or injury (41, 42). Accordingly, 4MU treatment resulted in a significant decrease in the number of IFNG^+^ and TNFA^+^ CD4^+^ T cells in the lungs, which was associated with a decrease in mRNA levels of type 1 markers *Ifng*, *Tnf*, and *Ccr5*. Accompanying these results, we also observed that 4MU-treated mice had decreased lung inflammation, decreased TUNEL^+^ cells, and improved pathology scores. We believe the improved outcomes in the treated mice were results of a reduced amount of LWM-HA.

4MU treatment, however, did not significantly modulate the number of CD8^+^ cells in the lungs between days 20 and 30. As CD8^+^ responses usually manifest 7–10 days after transplant and can account for greater than 90% of the immune response in the early stages of allograft rejection (43), these results do not preclude the possibility that CD8^+^ responses were well established before starting 4MU treatment and merits investigation if early administration of 4MU will modulate CD8^+^ responses. 

A key goal in allograft transplantation is the establishment of tolerance and graft acceptance. However, a major hurdle to achieving this is the presence of memory T cell responses. As memory T cells don’t require conventional costimulation (CD28), they can mount immediate secondary immune responses to pathogens or in the context of allograft transplantation, cross-react with donor antigens. Using transgenic CD8^+^ T cells, Maeshima et al. (44) demonstrated that in vitro–activated T cells were able to bind HA, and a small percentage of these cells gained a memory phenotype. Furthermore, these HA-binding memory cells increased over time following incubation with IL-7 or IL-15 and had greater proliferative capacity compared with T cells that did not bind HA. These data were also mirrored in vivo using OT-1 CD8^+^ T cells. This suggests that HAs binding to CD44^+^ memory T cells have a lower threshold for reactivation. In our model, we did not observe changes in the number of CD62^–^CD44^+^ effector T cells; we believe this was a result of administrating 4MU at a time point (day 20) in which T cell infiltration into the lungs is already well established and likely not amenable to change; however, 4MU did significantly decrease both CD4^+^ and CD8^+^ central memory T cells in the lung, suggesting that the use of 4MU may represent a new approach for promoting the induction of transplant tolerance.

Next, we investigated whether 4MU could directly block T cell activation. Canonically, T cell activation involves T cell receptor–MHC signaling, followed by costimulation between CD80/CD86 and CD28 (among many others) (45). However, there is evidence that HA signaling through CD44 signaling contributes to T cell priming, as well (46–48). In an in vitro study, it was demonstrated that activation of human T cells and IL-2 production could be potentiated using immobilized HA (49). Murine studies have shown that HAS1, HAS2, and HAS3 mRNAs are expressed by DCs and activated T cells, and upon using the HA inhibitor Pep-1, DC clusters were disrupted and proliferation of T cells was inhibited; furthermore, Pep-1 was also sufficient to block mitogen-induced T cell proliferation (50). These findings suggest that both T cells and DCs produce HA that contributes to T cell activation. Therefore, to examine the ability of 4MU to modulate T cell activation, we stimulated naive CD4^+^ T cells in vitro. When the T cells were concomitantly treated with 4MU, we observed a significant reduction in activated proliferating CD4^+^ T cells. A second finding from our in vitro assay was that naive T cells were also inhibited from being polarized into IFNG-producing Th1 type cells. These results extend the results from previous studies, highlighting the ability of 4MU to modulate T cell responses. In an in vitro study, 4MU reduced proliferation and IFNG production in mitogen-activated CD3^+^ T cells (29). In a diabetic mouse model, 4MU reduced HA deposits and CD3^+^ T cells in the pancreas and prevented progression of insulitis (30). 4MU also reduced infiltration of T cells into the kidneys and reduced the total number of CD4^+^IFNG^+^ cells in the spleens of lupus prone MRL/*lpr* mice (51). In the lungs of mice treated with LPS, 4MU reduced IFNG levels (52). Lastly, in murine models of experimental autoimmune encephalomyelitis, 4MU treatment reduced recruitment of Th1 cells into the spinal cord of diseased mice (53, 54). Collectively, our results — in addition to these studies — highlight that 4MU can modulate T cell responses in various disease models, including allograft rejection. To gain further insights into 4MU’s mechanism of action, we performed RNA-Seq of T cells stimulated and treated with 4MU. Not surprisingly, pathway analysis showed that signaling through the TLR is the most highly regulated pathway. HA signaling through the TLR has previously been shown to regulate lung injury (55) and promote chronic allograft rejection (24). Furthermore, TLRs are known modulators of immune cell activation (19, 56). In addition, GO pathways identified TNF as the most regulated pathway. This is in line with previous data showing that HA signaling through CD44 induces TNF expression through NF-κB–dependent pathways (20). These data offer compelling evidence, that 4MU is exerting effects through HA-dependent pathways; however, it is possible that some of the effects could be driven by non–HA-dependent pathways, and this possibility merits more investigation.

In summary, our results suggest that the use of 4MU represents a new approach to treat and reduce post–lung transplant rejection. 4MU is currently a clinically approved drug in Europe and, therefore, has a track record of safety in human use. Given our current findings, clinical studies examining 4MU in acute lung allograft rejection could have a significant impact on improving transplant tolerance.

## Methods

### Orthotopic lung transplantation.

Donor 10- to 12-week-old male BALB/c (allograft) (The Jackson Laboratory) mice were anesthetized with an i.p. injection of ketamine (87.5/kg) and xylazine (12.5 mg/kg) drug cocktail (38). The mice were then intubated and connected to a small animal ventilator (Harvard Apparatus). A median sternotomy was performed, and 100 U of heparin-sulfate was administered i.v. The lungs were flushed with saline, and the heart-lung was excised en block, followed by removal of the thymus. Cuffs were placed on the pulmonary artery, vein, and bronchus to isolate the donor left lung. The recipient C57BL/6 (The Jackson Laboratory) mice were similarly anesthetized, ventilated, and then underwent thoracotomy in the third intercostal space to retract their left lung lobe. The left pulmonary artery, vein, and bronchus of the recipient were then dissected free of the adjacent tissue, and the cuffed donor pulmonary artery, bronchus, and vein were inserted into corresponding recipient structures; a 10-0 nylon suture was used to perform an anastomosis between the donor lung and recipient. The recipient’s left lung was then excised out, and the incision site was closed and sutured together. C57BL/6 mice transplanted with isografts lung tissues were used as control mice in all animal experiments.

### 4MU drug therapy.

Starting at day 20 after transplant until day 30 after transplant, 450 mg/kg of 4MU (MilliporeSigma, catalog M1381) in 1% (or 5% where specified) Arabic gum (MilliporeSigma, catalog G9752) in PBS was orally administered daily to the mice that received allografts.

### In vitro T cell stimulation.

Spleen cells from C57BL/6 mice (The Jackson Laboratory) were prepared by rupturing the spleen in cold media (2% FBS in PBS) and filtering the suspension through a 40 μm cell strainer. The cells were washed twice, and naive CD4^+^ cells were isolated using an EasySep immunomagnetic negative selection kit (Stemcell Technologies, catalog 19765) following the manufacturer’s protocol. The isolated cells were stained with 5 μM CFSE (BioLegend, catalog423801) in PBS following the manufacturer’s protocol. In total, 500,000 CD4^+^ cells/well were then plated into 48-well tissue culture plates precoated with 10 μg/mL anti–mouse CD3E (BioLegend, catalog 100302) and 2 μg/mL anti–mouse CD28 (BioLegend, catalog 102102) in PBS. T cells were stimulated with 40 U/mL rhIL-2 (Roche, catalog 11011456001) with or without 4MU (100 μg/mL) (Sigma-Aldrich, catalog M1381-100G) in complete media (RPMI 1640 with 10% FBS, 1% penicillin/streptomycin, 50 μM 2-ME). On day 3 after stimulation, the supernatant was exchanged with fresh stimulation media with or without 4MU in the appropriate wells. The T cells were harvested on day 5 and analyzed using flow cytometry. For Th1 cell polarization, naive T cells were stimulated as described above with an additional 10 ng/mL rmIL-12 (R&D, catalog 419-ML) and 10 μg/mL anti–mouse IL-4 (BioLegend, catalog 504102) added to the media. On day 3 after stimulation, the supernatant was exchanged with fresh stimulation media with or without 4MU in the appropriate wells. On day 5 after stimulation, the cells were stimulated with 1× monensin (BioLegend, catalog 420701), 80 ng/mL PMA (Sigma-Aldrich, catalog P8139), and 1 μg/mL ionomycin (Sigma-Aldrich, catalog I9657) for 5 hours before collection and analysis by flow cytometry. Unstimulated naive T cells were cultured with complete media only in uncoated wells of the culture plate.

### Flow cytometry.

Donor left lungs were harvested from vehicle or 4MU-treated (5% Arabic gum in PBS) mice and minced using scissors. The lung pieces were placed in 5 mL of digestion media (RPMI 1640, 10% FBS, 1% PS, 400U/mL collagenase type IV, 50U/mL DNAse) on a shaker for 60 minutes at 37°C. The digested cells were filtered through a 40 μm cell strainer and washed twice by centrifuging the cells at 500*g* for 5 minutes at 4°C and suspending the pellet with cell media (RPMI 1640, 10% FBS, 1% penicillin/streptomycin). Cells were stimulated for 5 hours with 1× monensin (BioLegend, catalog 420701), 80 ng/mL PMA (Sigma-Aldrich, catalog P8139), and 1 μg/mL ionomycin (Sigma-Aldrich, catalog I9657). The cells were washed twice with cell staining buffer (1% BSA in PBS), incubated with rat anti–mouse CD16/CD32 (BioLegend, catalog 101301) (Supplemental Table 2) for 20 minutes on ice, and then directly stained anti-CD3E (BioLegend, catalog 100310), -CD4 (BioLegend, catalog 100536), and -CD8A (BioLegend, Catalog 100714) (Supplemental Table 2) for 30 minutes on ice. The cells were washed twice and then fixed and permeabilized with BD cytofix/cytoperm kit (BD Biosciences, catalog 554715) for 20 minutes following the manufacturers protocol. The cells were washed twice with 1× perm wash and then intracellularly stained for 30 minutes on ice with anti-TNFA (BioLegend, catalog 506304), -IFNG (BioLegend, catalog 505829), –IL-17A (BioLegend, catalog 506938), and –IL-4 (BioLegend, catalog 504104) in 1× perm wash (Supplemental Table 2). The cells were washed twice with 1× perm wash at 600*g* for 5 minutes at 4°C and then suspended in cell staining buffer. To profile memory T cells, the lung cells were surface stained against anti-CD4 (BioLegend, catalog 100528), -CD8A (BioLegend, catalog 100738), -CD62L (BioLegend catalog 104408), and -CD44 (BioLegend catalog 103006) (Supplemental Table 2). The stained lung cells were collected on a BD LSR Fortessa analyzer (BD Biosciences). In vitro–stimulated T cells harvested on day 5 were incubated with 1× monensin, 80 ng/mL PMA, and 1 μg/mL ionomycin for 5 hours and then washed twice with cell staining buffer, followed by surface staining with anti-CD3E (BioLegend, catalog 100310), -CD4 (BioLegend, catalog 100536), -CD25 (BioLegend, catalog 101921), -CD69 (BioLegend, catalog 104512), and -CD279 (PD1) (BioLegend, catalog 135209). The cells were then fixed and permeabilized and intracellularly stained with anti-IFNG (BioLegend, catalog 505829) (Supplemental Table 2). The cells were washed twice 1× perm wash and then suspended in cell staining buffer. The cells were collected on a BD LSR Fortessa cell analyzer (BD Biosciences). All data were analyzed using FlowJo (Tree Star Inc.), with Fluorescence Minus One control samples used to draw positive and negative gates.

### Histological staining.

The donor lung was dissected, and the distal portion was fixed in formalin for 48 hours. The lungs were then embedded in paraffin wax, cut into 5 μm sections, and stained with H&E (MilliporeSigma, catalog MHS32-1L) and Masson’s trichrome (MilliporeSigma, catalog HT15). The stained lungs were captured at 200× magnification on an Olympus VS120 scanning microscope (Olympus Corporation). Lung Pathology scores were blindly evaluated by a pulmonologist using the criteria outlined (57, 58).

Left lungs were stained with biotinylated HABP (Seikagaku Ltd., catalog 400763), followed by Texas Red–conjugated streptavidin secondary stain (Jackson ImmunoResearch Laboratories Inc.). Stained sections were captured on a VS120 virtual slide microscope (Olympus Corporation). To confirm the specificity of HA staining, serial sections of lung tissue were treated with hyaluronidase (Seikagaku Ltd., catalog 100740-1) before HA staining (not shown). The total fluorescent intensity of each lung was then calculated using Fiji ImageJ (NIH) and divided by the area of the lung.

### IHC.

Whole lung sections were stained with rat anti–mouse CD4 (Invitrogen, catalog 14-9766-80) and rat anti–mouse CD8A (Invitrogen, catalog 14-0808-82) (Supplemental Table 2) in 0.1% BSA, 0.1% Triton-x overnight. The sections were then stained with anti–rat HRP-DAB cell and tissue staining kit following the manufacturer’s protocol (R&D, CTS017), followed by counterstain with Mayers Hematoxylin (MilliporeSigma, MHS32-1L). Stained sections were then captured at 200× magnification using a VS120 slide scanning microscope (Olympus Corporation). DAB^+^ cells were then quantified from 5 random high-powered fields (200× magnification) per lung section using Fiji ImageJ. 

### HA Molecular size analysis.

The molecular size of HA was measured as previously described (59–61). Briefly, pooled tissue samples (*n* = 5/group; total weight, 60 mg) from the left lung were digested with proteinase K and chondroitinase ABC (pH 7.0). The digested samples, along with molecular weight ladders (Hyalose LLC), were loaded onto a 0.7% agarose gel and then stained with Stain-All (Sigma-Aldrich) following electrophoresis. Gel images were then captured using ChemiDoc XRS^+^ and analyzed with Quantity One software (Bio-Rad).

### TUNEL staining.

Whole lung sections (5 μm) were stained for apoptotic cells using a TUNEL staining kit (Takara Bio, catalog MK500) following the manufacturer’s instructions. Stained sections were mounted with DAPI mounting media (Vector Labs) and then captured at 200× magnification using a Nikon Eclipse 80i microscope (Nikon). TUNEL^+^ and DAPI^+^ cells were then quantified and merged using Fiji ImageJ (NIH).

### RNA extraction and qPCR.

Total RNA was extracted from the upper portion of the left lung tissue using the RNeasy mini kit (Qiagen, catalog74104) following the manufacturer’s protocol. Equal mounts of RNA were reverse transcribed into cDNA using the AmplifiRivert Reverse transcription kit (GenDEPOT, catalogR5101) following the manufacturer’s protocol. In total, 1 μL of cDNA was used for qPCR using a qPCR master mix kit (Qiagen, catalog 330529) using a Real-Time PCR machine (Bio-Rad). Kickstart SYBR green primers were synthesized by Sigma-Aldrich and are listed in Supplemental Table 3.

### RNA-Seq, differential expression, and pathway analysis.

RNA from in vitro stimulated naive CD4 T cells was extracted using Qiagen RNA easy extraction Kit following the manufacture’s protocol (Qiagen, catalog 74104). cDNA synthesis was performed using the SMART-Seq v4 Ultra Low Input RNA Kit for Sequencing and quantified using Qubit. Libraries were constructed using the Covaris system and quantified using a Qubit 2.0 fluorometer. Libraries were pooled and sequenced using Illumina machines based on activity and expected data volume. FASTQ files were aligned to BAM files using the Rsubread package (62, 63) and the GENCODE (release M27/GRCm39) mouse reference genome. Count matrices were also generated using Rsubread. Before analysis, genes with fewer than 1 count per million in > 3 samples were excluded. Differential expression was performed using the limma and voom packages, where associations between expression levels and treatment group were tested. Given the sample size, no other covariates were included. Multiple testing was controlled for a FDR < 10%. Pathway analysis was conducted using the fgsea package, with reference pathway gene sets from the KEGG canonical pathways, GO biological processes pathways, and GO molecular function pathways. Pathways were considered significantly enriched if nominal *P* < 0.05. Heatmaps were constructed using log-transformed counts and the pheatmap package. All statistical analyses were performed in R (v4.1.0).

### Data availability.

The RNA-Seq data supporting the findings of this study are available in GEO DATABASE at https://www.ncbi.nlm.nih.gov/geo/ (GSE183142). All other data supporting the findings of this study are available within the article and Supplemental Methods.

### Statistics.

Data herein are presented as mean ± SEM. Where vehicle- and 4MU-treated groups were compared, a 2-tailed Student’s *t* test was used; otherwise, 1-way ANOVA, followed by Tukey’s post hoc test, was used to compare control, vehicle-treated, and 4MU-treated groups. Grubbs test for outliers was used to determine outlier values from all data sets. Statistical analyses were performed using GraphPad Prism 9.02 (GraphPad Software). A *P* value of less than 0.05 was considered significant.

### Study approval.

The study design and use of animals for this manuscript was reviewed and approved by the IACUC at Boston Children’s Hospital (IACUC protocol no. 19-10-3997R)

## Author contributions

The creation of this manuscript relied on the contribution of multiple authors. The research questions and studies were conceptualized by JI, YC, JA, GV, and SEC and were conducted by JI, KL, YC, JPA, JH, JN, SS, AML, PHL, AH, and AJE. The data were acquired by JI, YC, JPA, and XL and analyzed by JI, YC, AJG, IOR, MAP, JA, GV, and SEC. The manuscript was written by JI and SEC, and it was approved by all the contributing authors.

## Supplementary Material

Supplemental data

## Figures and Tables

**Table 1:**
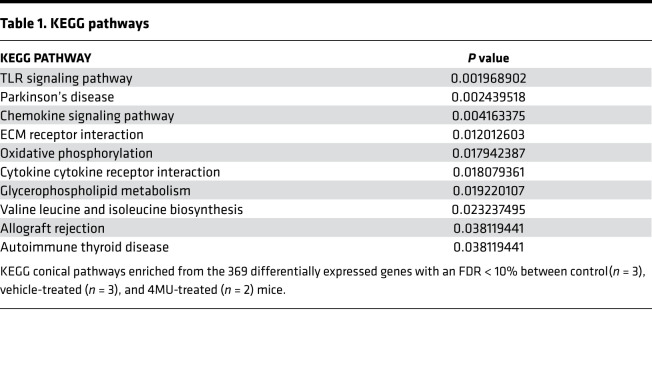
KEGG pathways

## Version 1 10/19/2021

In-Press Preview

## Version 2 11/22/2021

Electronic publication

## References

[1] Yeung JC, Keshavjee S (2014). Overview of clinical lung transplantation. Cold Spring Harb Perspect Med.

[2] Chambers DC (2018). The International Thoracic Organ Transplant Registry of the International Society for Heart and Lung Transplantation: thirty-fifth adult lung and heart-lung transplant report-2018; focus theme: multiorgan transplantation. J Heart Lung Transplant.

[3] He H (2002). Analysis of robust innate immune response after transplantation in the absence of adaptive immunity. Transplantation.

[4] He H (2003). Analysis of differential immune responses induced by innate and adaptive immunity following transplantation. Immunology.

[5] Ridge JP (1998). A conditioned dendritic cell can be a temporal bridge between a CD4+ T-helper and a T-killer cell. Nature.

[6] Celli S (2011). Visualizing the innate and adaptive immune responses underlying allograft rejection by two-photon microscopy. Nat Med.

[7] Weigel PH, DeAngelis PL (2007). Hyaluronan synthases: a decade-plus of novel glycosyltransferases. J Biol Chem.

[8] Itano N (2002). Abnormal accumulation of hyaluronan matrix diminishes contact inhibition of cell growth and promotes cell migration. Proc Natl Acad Sci U S A.

[9] Stern R (2006). Hyaluronan fragments: an information-rich system. Eur J Cell Biol.

[10] Fallacara A (2018). Hyaluronic acid in the third millennium. Polymers (Basel).

[11] Stern R, Jedrzejas MJ (2006). Hyaluronidases: their genomics, structures, and mechanisms of action. Chem Rev.

[12] Knudson CB, Knudson W (1993). Hyaluronan-binding proteins in development, tissue homeostasis, and disease. FASEB J.

[13] Ruffell B (2011). Differential use of chondroitin sulfate to regulate hyaluronan binding by receptor CD44 in inflammatory and interleukin 4-activated macrophages. J Biol Chem.

[14] Jiang D (2007). Hyaluronan in tissue injury and repair. Annu Rev Cell Dev Biol.

[15] Ruppert SM (2014). Tissue integrity signals communicated by high-molecular weight hyaluronan and the resolution of inflammation. Immunol Res.

[16] Jiang D (2011). Hyaluronan as an immune regulator in human diseases. Physiol Rev.

[17] Tesar BM (2006). The role of hyaluronan degradation products as innate alloimmune agonists. Am J Transplant.

[18] Powell JD, Horton MR (2005). Threat matrix: low-molecular-weight hyaluronan (HA) as a danger signal. Immunol Res.

[19] Termeer C (2002). Oligosaccharides of hyaluronan activate dendritic cells via toll-like receptor 4. J Exp Med.

[20] McKee CM (1996). Hyaluronan (HA) fragments induce chemokine gene expression in alveolar macrophages. The role of HA size and CD44. J Clin Invest.

[21] Horton MR (1998). Regulation of hyaluronan-induced chemokine gene expression by IL-10 and IFN-gamma in mouse macrophages. J Immunol.

[22] Yamawaki H (2009). Hyaluronan receptors involved in cytokine induction in monocytes. Glycobiology.

[23] de la Motte C (2009). Platelet-derived hyaluronidase 2 cleaves hyaluronan into fragments that trigger monocyte-mediated production of proinflammatory cytokines. Am J Pathol.

[24] Todd JL (2014). Hyaluronan contributes to bronchiolitis obliterans syndrome and stimulates lung allograft rejection through activation of innate immunity. Am J Respir Crit Care Med.

[25] Cui Y (2015). Therapeutic lymphangiogenesis ameliorates established acute lung allograft rejection. J Clin Invest.

[26] Nagy N (2010). Inhibition of hyaluronan synthesis accelerates murine atherosclerosis: novel insights into the role of hyaluronan synthesis. Circulation.

[27] Rilla K (2004). The hyaluronan synthesis inhibitor 4-methylumbelliferone prevents keratinocyte activation and epidermal hyperproliferation induced by epidermal growth factor. J Invest Dermatol.

[28] Kakizaki I (2004). A novel mechanism for the inhibition of hyaluronan biosynthesis by 4-methylumbelliferone. J Biol Chem.

[29] Mahaffey CL, Mummert ME (2007). Hyaluronan synthesis is required for IL-2-mediated T cell proliferation. J Immunol.

[30] Nagy N (2015). Inhibition of hyaluronan synthesis restores immune tolerance during autoimmune insulitis. J Clin Invest.

[31] Kultti A (2009). 4-Methylumbelliferone inhibits hyaluronan synthesis by depletion of cellular UDP-glucuronic acid and downregulation of hyaluronan synthase 2 and 3. Exp Cell Res.

[32] Yoshihara S (2005). A hyaluronan synthase suppressor, 4-methylumbelliferone, inhibits liver metastasis of melanoma cells. FEBS Lett.

[33] Krupnick AS (2009). Orthotopic mouse lung transplantation as experimental methodology to study transplant and tumor biology. Nat Protoc.

[34] Miller ML (2017). Transplantation tolerance after allograft rejection. Curr Opin Organ Transplant.

[35] Espinosa JR (2016). Memory T cells in organ transplantation: progress and challenges. Nat Rev Nephrol.

[36] Gelman AE (2008). CD4+ T lymphocytes are not necessary for the acute rejection of vascularized mouse lung transplants. J Immunol.

[37] Boisgérault F (2001). Role of CD4+ and CD8+ T cells in allorecognition: lessons from corneal transplantation. J Immunol.

[38] Liu K (2014). Inhibition of the purinergic pathway prolongs mouse lung allograft survival. Am J Respir Cell Mol Biol.

[39] Lendermon EA (2015). CD8(+)IL-17(+) T cells mediate neutrophilic airway obliteration in T-bet-deficient mouse lung allograft recipients. Am J Respir Cell Mol Biol.

[40] Kreisel D (2002). Non-hematopoietic allograft cells directly activate CD8+ T cells and trigger acute rejection: an alternative mechanism of allorecognition. Nat Med.

[41] DeGrendele HC (1997). Requirement for CD44 in activated T cell extravasation into an inflammatory site. Science.

[42] Siegelman MH (1999). Activation and interaction of CD44 and hyaluronan in immunological systems. J Leukoc Biol.

[43] Braun MY (2001). Acute rejection in the absence of cognate recognition of allograft by T cells. J Immunol.

[44] Maeshima N (2011). Hyaluronan binding identifies the most proliferative activated and memory T cells. Eur J Immunol.

[45] Smith-Garvin JE (2009). T cell activation. Annu Rev Immunol.

[46] Do Y (2004). Role of CD44 and hyaluronic acid (HA) in activation of alloreactive and antigen-specific T cells by bone marrow-derived dendritic cells. J Immunother.

[47] Ariel A (2000). Induction of interactions between CD44 and hyaluronic acid by a short exposure of human T cells to diverse pro-inflammatory mediators. Immunology.

[48] Huet S (1989). CD44 contributes to T cell activation. J Immunol.

[49] Galandrini R (1994). Hyaluronate is costimulatory for human T cell effector functions and binds to CD44 on activated T cells. J Immunol.

[50] Mummert ME (2002). Synthesis and surface expression of hyaluronan by dendritic cells and its potential role in antigen presentation. J Immunol.

[51] Suarez-Fueyo A (2019). Hyaluronic acid synthesis contributes to tissue damage in systemic lupus erythematosus. Front Immunol.

[52] McKallip RJ (2015). Treatment with the hyaluronic acid synthesis inhibitor 4-methylumbelliferone suppresses LPS-induced lung inflammation. Inflammation.

[53] Kuipers HF (2016). Hyaluronan synthesis is necessary for autoreactive T-cell trafficking, activation, and Th1 polarization. Proc Natl Acad Sci U S A.

[54] Mueller AM (2014). Inhibition of hyaluronan synthesis protects against central nervous system (CNS) autoimmunity and increases CXCL12 expression in the inflamed CNS. J Biol Chem.

[55] Jiang D (2005). Regulation of lung injury and repair by Toll-like receptors and hyaluronan. Nat Med.

[56] Kawasaki T, Kawai T (2014). Toll-like receptor signaling pathways. Front Immunol.

[57] Stewart S (2007). Revision of the 1996 working formulation for the standardization of nomenclature in the diagnosis of lung rejection. J Heart Lung Transplant.

[58] Martinu T (2019). Spectrum of chronic lung allograft pathology in a mouse minor-mismatched orthotopic lung transplant model. Am J Transplant.

[59] Lee HG, Cowman MK (1994). An agarose gel electrophoretic method for analysis of hyaluronan molecular weight distribution. Anal Biochem.

[60] van der Windt GJW (2010). CD44 deficiency is associated with increased bacterial clearance but enhanced lung inflammation during Gram-negative pneumonia. Am J Pathol.

[61] Forteza RM (2012). Hyaluronan and layilin mediate loss of airway epithelial barrier function induced by cigarette smoke by decreasing E-cadherin. J Biol Chem.

[62] Liao Y (2013). The Subread aligner: fast, accurate and scalable read mapping by seed-and-vote. Nucleic Acids Res.

[63] Liao Y (2014). featureCounts: an efficient general purpose program for assigning sequence reads to genomic features. Bioinformatics.

