# Supplementary material for: Blocking hyaluronan synthesis alleviates acute lung allograft rejection
Source: JCI Insight. 2021 Nov 22;6(22):e142217. doi: 10.1172/jci.insight.142217 (PMC8663774; doi:10.1172/jci.insight.142217)
Supplement: Supplemental data [file jciinsight-6-142217-s032.pdf]

Supplementary Material

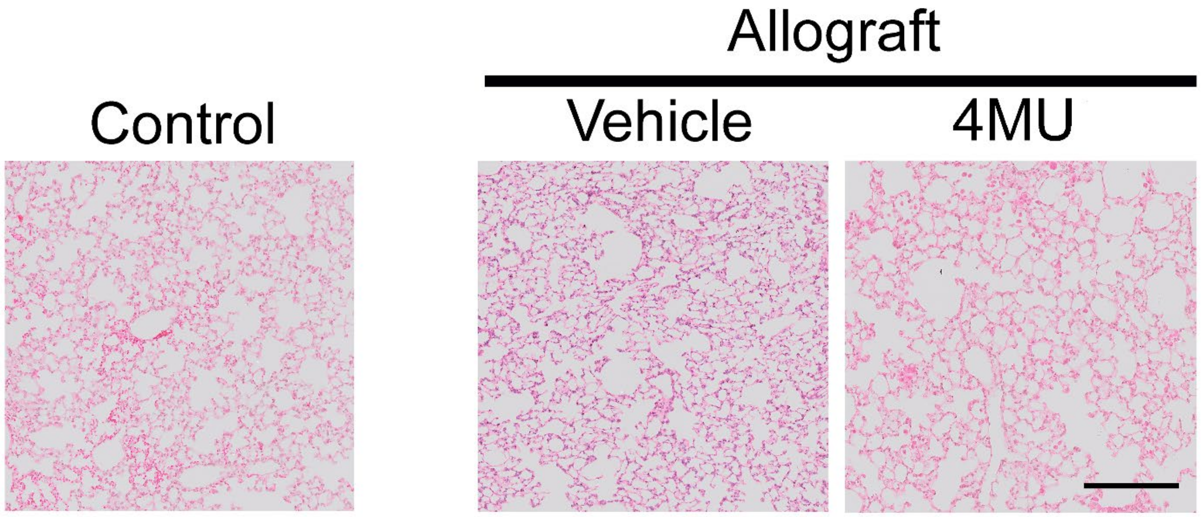

Supplement Figure 1 - Right Lungs of Recipient Mice
Representative images of right lung sections from indicated recipient mice stained with H&E imaged at 200x magnification (scale bar 200 µm).

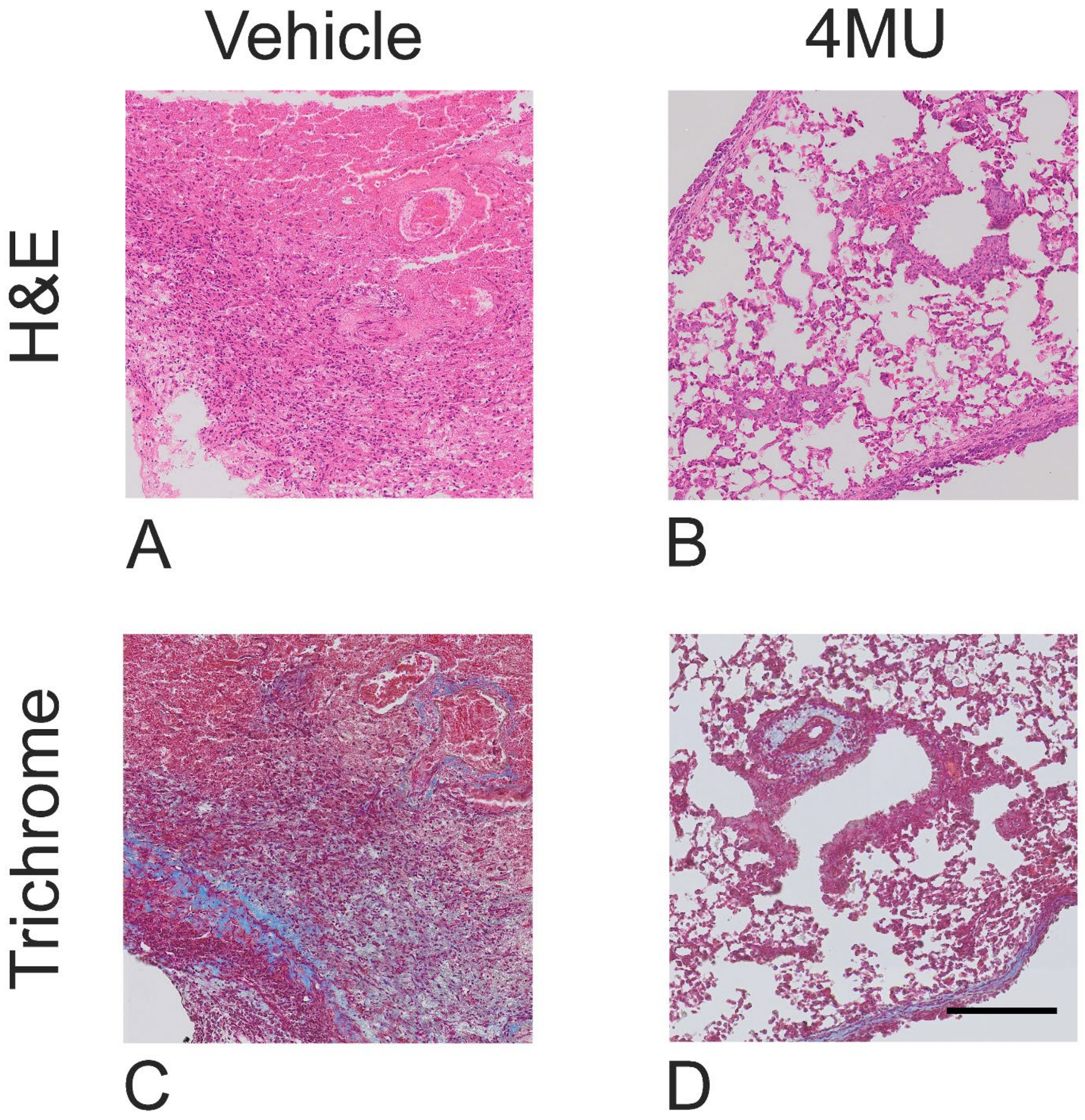

Supplement Figure 2 – Histological Features of Lungs following Allogeneic Transplantation
Representative images from indicated recipient mice stained with H&E and Masson's Trichome and imaged at 200x (scale bar 200 µm). Untreated mice with severe lung injury (ACR) characterized by inflammation (A) and diffuse parenchymal fibrosis with thick pleuritis with fibrosis (C). Allograft recipients treated with 4MU had reduced ACR (B) with little peri bronchial fibrosis, mild pleuritis with fibrosis, and no parenchymal fibrosis (D).

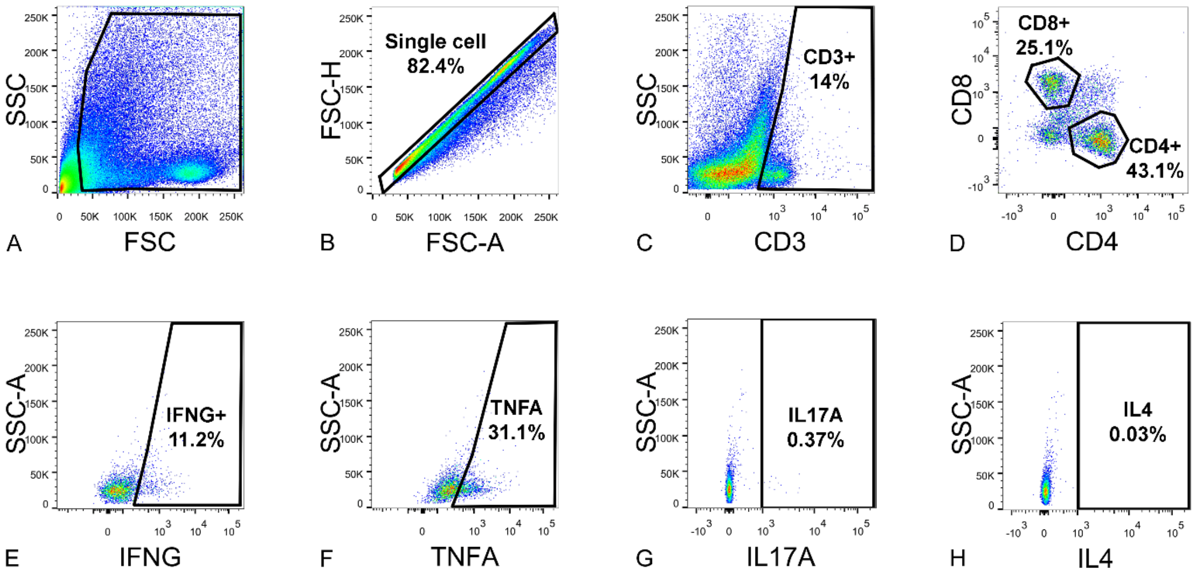

Supplement Figure 3 – Flow Cytometry gating strategy of Stained Lung Cells.
Single suspension of donor lung cells were stained with anti CD3E, CD4, CD4, CD8A, IFNG, TNFA, IL17A, and IL4. Cells were gated to remove debris (A) and doublet cells (B) followed by CD3 (C) and CD4 (D). IFNG(E), TNFA(F), IL17A(G), and IL4(H) were individually gated from the CD4 parent population.

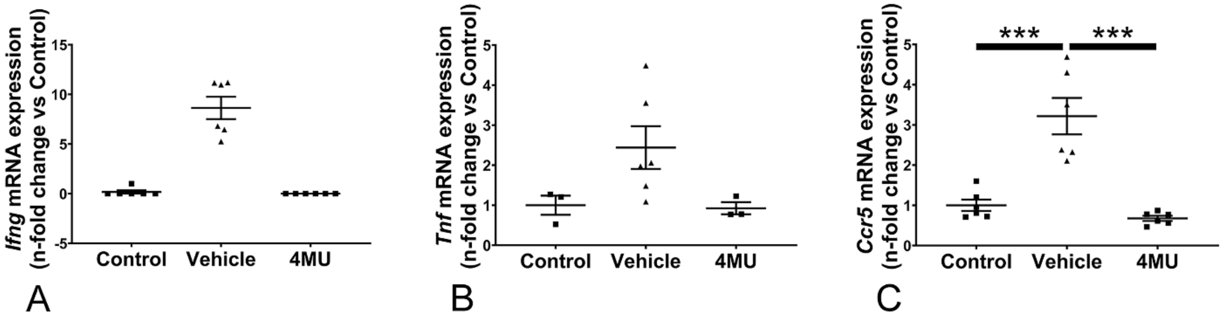

Supplement Figure 4 - mRNA from donor left lungs.
RNA was extracted from donor left lung lysates of control(n6), vehicle(n6) and 4-MU(n6) treated mice and reverse transcribed to cDNA followed by qPCR analysis of mRNA expression of type 1 markers: *Ifng* (A), *Tnf* (B), and *Ccr5* (C). Data represented as mean ± SEM and analyzed using one-way ANOVA followed by Tukey's post-hoc test for multiple comparisons, *** $p<0.001$, **** $p<0.0001$.

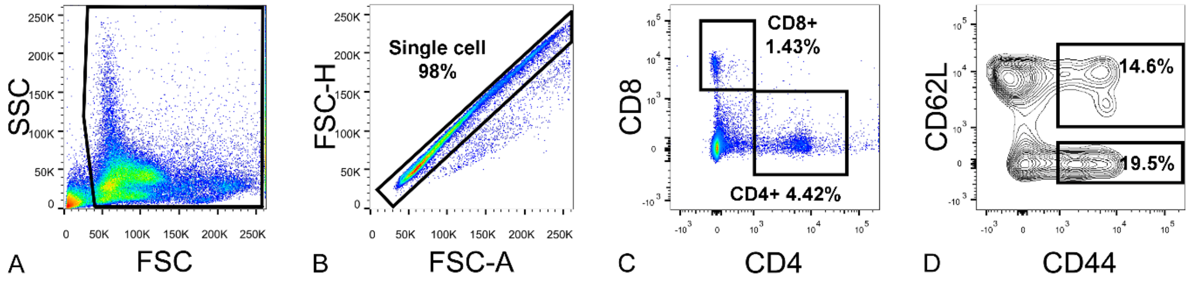
SSC
FSC
A
FSC-H
FSC-A
Single cell
98%
B
CD8
CD4
CD8+
1.43%
CD4+ 4.42%
C
CD62L
CD44
14.6%
19.5%
D

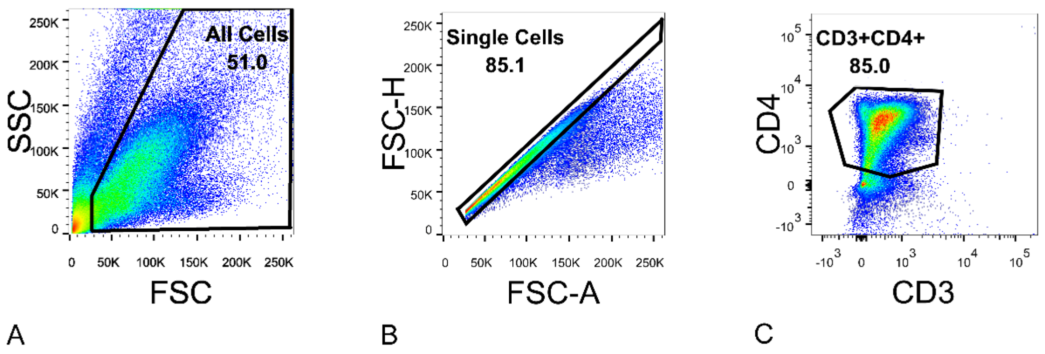

Supplement Figure 6 - Flow Cytometry gating strategy of stimulated CFSE labeled C57BL/6 Cells.
Naïve CD4 T-cells (n3) stimulated in vitro with CD3E, CD28, rhIL-2, and treated with 4MU. Cells were stained with anti CD3E, CD4, CD25, CD69, CD279. Stimulated T-cells were gated to exclude debris (A) and doublet cells (B) followed by a gate to include both CD3+ and CD4+ cells (C). These cells were then independently gated for CD25+, CD69+, CD279+, or CFSE expression.

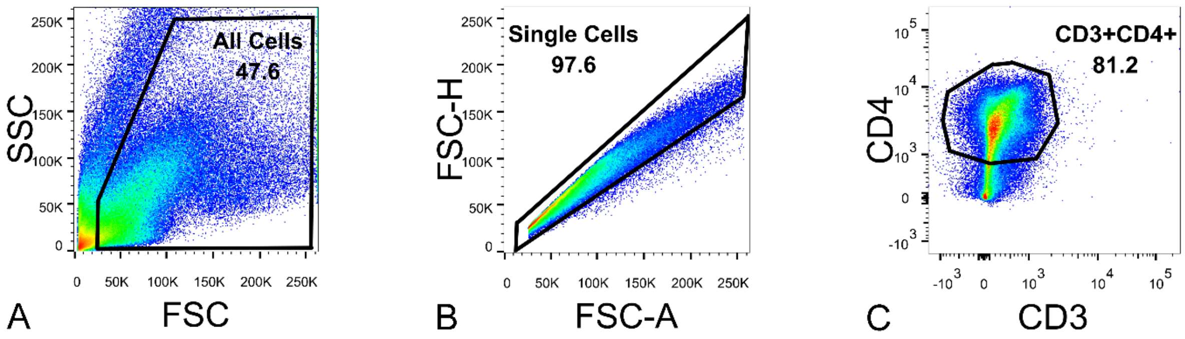

Supplement Figure 7 - Flow Cytometry gating strategy of Th1 polarized C57BL/6 cells
Naïve CD4 T-cells (n3) stimulated with CD3E, CD28, rhIL-2, rmIL-12, anti-IL-4, and treated with 4MU. Cells were stained with anti CD3E, CD4, & IFNG. Cellular debris (A) and doublet cells (B) were gated out followed by a gate to include both CD3+ and CD4+ cells (C). These cells were then independently gated for IFNG and CFSE expression.

GO: BP Pathways		
Pathway	p value	NES
RESPONSE TO TUMOR NECROSIS FACTOR	6.59E-05	2.223380934
NEGATIVE REGULATION OF DEVELOPMENTAL GROWTH	9.53E-05	1.702466543
POSITIVE REGULATION OF ION TRANSPORT	0.000118756	2.387683663
REGULATION OF CHEMOTAXIS	0.000156073	2.174049874
LYMPHOCYTE CHEMOTAXIS	0.000250349	1.89736613
MONOCYTE CHEMOTAXIS	0.000250349	1.89736613
ENERGY COUPLED PROTON TRANSMEMBRANE TRANSPORT AGAINST ELECTROCHEMICAL GRADIENT	0.000256486	-1.859901877
POSITIVE REGULATION OF CHEMOTAXIS	0.000392742	2.060475084
MULTICELLULAR ORGANISMAL HOMEOSTASIS	0.000445485	2.101521799
NATURAL KILLER CELL CHEMOTAXIS	0.001052746	1.669905707

Supplement Table 1 – GO: BP Pathways
Top 10 Gene Ontology Biological processes pathways derived from the top 369 differentially regulated genes with an FDR <10% between control (n3), vehicle (n3), and 4MU (n2) treated mice. NES: normalized enrichment score

Supplement Table 2 – List of Antibodies

List of Antibodies used for IHC and flow cytometry staining.

Target	Conjugate	Host	Vendor
CD3E	PE-Cy5	Hamster	Biolegend Cat# 100310 (145-2C11)
CD4	Alexa Flour 700<br>PE-Cy7<br>IHC unconjugated	Rat<br>Rat<br>Rat	Biolegend Cat# 100536 (RM4-5)<br>Biolegend Cat#100528 (RM4-5)<br>Thermofisher Cat# 14-9766-80 (4SM95)
CD8A	BV421<br>IHC Unconjugated	Rat<br>Rat	Biolegend Cat#100738 (53-6.7)<br>Thermofisher Cat# 14-0808-82 (4SM15)
CD25	APC/Fire 750	Rat	Biolegend Cat# 101921 (3C7)
CD44	FITC	Rat	Biolegend Cat#103006 (IM7)
CD62L	PE	Rat	Biolegend Cat#104408 (MEL-14)
CD69	PE-Cy7	Hamster	Biolegend Cat# 104512 (H1.2F3)
CD279	APC	Rat	Biolegend Cat# 135209 (29F.1A12)
IFNG	BV421	Rat	Biolegend Cat# 505829 (XMG1.2)
TNFA	FITC	Rat	Biolegend Cat# 506304 (MP6-XT22)
IL4	PE	Rat	Biolegend Cat# 504104 (11B11)
IL17A	PE-Dazzle-594	Rat	Biolegend Cat# 506938 TC11-18H10.1)
CD16/CD32	Unconjugated	Rat	Biolegend Cat# 101301 (93)

Supplement Table 3 - PCR Primers

List of sense and Anti-sense PCR Primers used to amplify mRNA from lung lysates

Target Gene	Primer Sequence 5’ to 3’
Ccr5	Sense – AGACCTAAATCCTACCACAC<br><br>Antisense – TGAGTATTGCCAAGTTTGAG
Ifng	Sense - TTGAAAGCCTAGAAAGTCTG<br><br>Antisense - AAAGAGTCTGAGGTAGAAAGAG
Tnf	Sense - CTATGTCTCAGCCTCTTCTC<br><br>Antisense – CATTTGGGAACTTCTCATCC
Gapdh	Sense - TGTGTCCGTCGTGGATCTGA<br><br>Antisense – CCTGCTTCACCACCTTCTTGA
B2m	Sense - GTATGCTATCCAGAAAACCC<br><br>Antisense - CTGAAGGACATATCTGACATC
